# For Debate: The 2023 European Society of Hypertension guidelines - cause for concern

**DOI:** 10.1097/HJH.0000000000003733

**Published:** 2024-04-12

**Authors:** Eduard Shantsila, D. Gareth Beevers, Gregory Y.H. Lip

**Affiliations:** a Department of Primary Care and Mental Health, University of Liverpool; b Liverpool Centre for Cardiovascular Science at University of Liverpool, Liverpool John Moores University and Liverpool Heart & Chest Hospital, Liverpool; c University of Birmingham Department of Medicine, City Hospital, Birmingham, UK; d Danish Center for Health Services Research, Department of Clinical Medicine, Aalborg University, Aalborg, Denmark

**Keywords:** beta-blockers, guidelines, hypertension

## Abstract

Originally, the beta-blockers were equally ranked alongside the other antihypertensive drug classes. Things changed when two major long-term randomized controlled trials, ASCOT-BPLA and LIFE showed that the patients receiving the beta-blockers based regimes suffered 25–30% more strokes than those receiving a calcium channel blocker based regime or an angiotensin receptor blocker based regime. The inferiority of the beta-blockers at stroke prevention was not due to differences in blood pressure control during the follow-up period in both trials. The 2023 European Society of Hypertension (ESH) guidelines still argue in favour of beta-blockers that their clinical inferiority was simply to lesser blood pressure reduction rather than class effect. The analysis argues that the return of beta-blockers as a first-line option for the management of uncomplicated hypertension by the ESH is a cause for concern and should be reconsidered.

Hypertension affects millions worldwide, and many organizations and learned societies have produced guidelines for its optimal management. Most agree that the best first-line therapy, when drug treatment is considered necessary, are the calcium channel blockers (CCBs), the angiotensin blockers or the thiazide-type diuretics.

Beta-blockers have long been considered best reserved for patients with concomitant comorbidities, where they have been shown to be effective. For example, they are used in patients with paroxysmal or persistent atrial fibrillation or symptomatic coronary heart disease. Also, they are indicated in patients who have recovered from a myocardial infarction or have heart failure.

Originally, the beta-blockers were equally ranked alongside the other drug classes. Things changed when two major long-term randomized controlled trials (RCTs), ASCOT-BPLA and LIFE showed that the patients receiving the beta-blocker based regimes suffered 25–30% more strokes than those receiving a CCB-based regime or an angiotensin receptor blocker (ARB) based regime [1–3]. It is important to note that the inferiority of the beta-blockers at stroke prevention was not due to differences in blood pressure (BP) control during the follow-up period in both trials.

The independent investigator-led ASCOT-BPLA RCT compared amlodipine, with perindopril added if further BP reduction was required, (*n* = 9639) or atenolol with bendroflumethiazide added as necessary (*n* = 9618) [2]. The study was stopped early after a median 5.5-year follow-up due to 23% lower rates of stroke (3.4 vs. 4.4%), 11% lower mortality (7.7 vs. 8.5%) and 16% lower rates of total cardiovascular events and procedures (14.1 vs. 16.7%) in the amlodipine-based group. Of note, patients in the amlodipine group had a 30% lower risk of developing diabetes (11 vs. 16%), 35% fewer cases of peripheral artery disease (1.4 vs. 2.1%) and a 15% lower risk of renal impairment (4.2 vs. 4.9%, *P* < 0.05 for all). There was no difference in life-threatening arrhythmias, thus not demonstrating the benefits of this population's heart rate limiting and antiarrhythmic properties of BBs.

In the LIFE RCT, patients with hypertension and ECG criteria of left ventricular hypertrophy were randomized to losartan-based (*n* = 4605) or atenolol-based (*n* = 4588) treatment with a mean follow-up of 4.8 years [1]. The risk of the primary endpoint (death, myocardial infarction or stroke) was 13% lower (11 vs. 13%), and the risk of stroke was 25% lower (5.0 vs. 6.7%) in the losartan group. The two trials leave no doubt about the inferiority of beta-blocker based treatments.

The question of drug side effects must also be faced. The CCBs, the ARBs and the thiazide diuretics have few side effects if used in low doses. The angiotensin-converting enzyme inhibitors (ACE-I) cause an irritating dry cough, especially at night. In a recent systematic review of 99 RCTs, 8% of patients received ACE-I vs. 4% in the placebo arm [4]. Although the cough typically needs ACE-I discontinuation, it is reversible. ACE-I may cause potentially life-threatening angioedema, even though a meta-analysis (12 RCTs, *n* = 33 337) did not find a significant overall difference compared with placebo in angioedema incidence vs. placebo [4]. ARBs generally have fewer of these side effects and are good alternatives, which makes side effects concerns about beta-blockers even more stark.

Side effect profile is a major weakness of beta-blockers when used for BP control. In the ASCOT-BPLA trial, more patients stopped atenolol-based therapy than amlodipine-based therapy (3 vs. 2%, *P* < 0.0001) [2]. Beta-blocker based treatment was associated with a two-fold higher rate of fatigue (16%), more bradycardia, chest pain, dizziness, dyspnoea, erectile dysfunction and lethargy (*P* < 0.0001 for all). Similarly, in the LIFE trial, there was a 25% lower incidence of new-onset diabetes (5.2 vs. 7.0%) in the losartan group than atenolol. Patients in the atenolol group have more bradycardia, dyspnoea, fatigue, sexual dysfunction, episodes of hypotension, albuminuria, leg oedema and more episodes of pneumonia [1].

When the beta-blockers became available in about 1965, they were considered a significant breakthrough as they were much more tolerable than the drugs then in use (the adrenergic neuron blockers such as bethanidine or methyldopa). Since then, it has become apparent that the beta-blockers were associated with an insidious onset of lethargy and reduced exercise tolerance. Patients also noticed sleep disturbance [5]. There was also more sexual dysfunction in the beta-blocker arm in both trials. An obvious excess in new diabetes in patients receiving beta-blockers for hypertension control is a significant concern, which may explain their clinical inferiority to CCB and ARBs. Although newer generations of beta-blockers may be safer, clinical trials evidence of their use as first-line agents for hypertension, including those with physiological higher rates, are lacking. Recommending them based on pathophysiological considerations and limited observational studies may put patients at risk.

The 2023 European Society of Hypertension (ESH) guidelines argue in favour of beta-blockers that their clinical inferiority was simply to lesser BP reduction achieved in the RCT rather than class effect. BP values were lower throughout the ASCOT-BPLA trial in the amlodipine-based group, with an average difference of 2.7/1.9 mmHg. In the LIFE study, BP fell by 30.2/16.6 mmHg in the losartan and 29.1/16.8 mmHg in the atenolol group (*P* = 0.017 for SBP, NS for DBP). Even if lesser BP-lowering effects of beta-blockers played a role, this would still be another disadvantage. Every 5 mmHg reduction in SBP reduces major cardiovascular events by 10%, even at normal BP, making undertreatment rather than overtreatment of hypertension a major concern, especially in younger adults [6].

Virtually, all patients in RCTs have required a combination of drugs to control hypertension to target [7]. BP is appropriately controlled in less than half of treated real-world patients, and the true number of medications needed to control BP is hard to establish accurately [8]. The ESC Hypertension guidelines [9] highlight the following key reasons for failure to achieve BP control: physician inertia to adequately up-titrate treatment [10], low patient adherence to treatment (<50% based on prescription refilling) [11], higher rates of treatment nonadherence with more medications used [12]. This points out the importance of using well tolerated drugs that are more likely to achieve BP control, with beta-blockers failing to meet the criteria.

There is a further concern about the safety of beta-blockers in older people, especially if they develop frailty. In people aged 80 years and older, beta-blockers have been associated with postural hypotension, which could be attributed to impaired cardiac chronotropic and inotropic function due to ageing-related myocardial fibrosis [13]. In the HYVET trial of octogenarians, beta-blockers were the only class of antihypertensive drugs independently associated with the risk of fractures [hazard ratio 2.05, 95% confidence interval (95% CI) 1.03–4.08] [14].

Given this evidence, it seems strange that the new 2023 ESH guidelines reinstate the beta-blockers as suitable first-line therapy for hypertension, although with caveats in other paragraphs. Why is this volte-face based on no new evidence? The message is confusing, and we profoundly disagree. We are not alone in these views; Messerli, Bangalore and Mandrola share many of our sentiments in a similarly detailed review [15].

In a very long guideline manuscript, it is easy to miss the text that describes the lack of trial evidence supposing first-line use of beta-blockers for new indications and downplays the evidence of their inferiority for stroke prevention, a complication that matters. Guidelines should be short, sharp and easy to read so that busy clinicians can use them when seeing their next patient to inform joint decisions supported by evidence.

Sadly, all the guideline committees (from the USA, the UK and Europe) largely comprise hospital-based specialists. In contrast, most patients with hypertension are managed exclusively in primary care settings because they are less likely to have cardiovascular complications of hypertension. Hospital-based hypertension specialists are more likely to encounter more complex cases many of whom may have suffered the vascular complications of hypertension. The re-endorsement of the beta-blockers for uncomplicated hypertension might not have happened if more primary care physicians had had greater input into the preparation of the guidelines.

To some extent, arguments about first-line drugs are of only limited use as, in most patients, double or triple therapy is necessary to bring the BP under control. But even then, the beta-blockers are only required if there is concomitant heart disease. In primary healthcare, such patients are in the minority. Hence, the return of beta-blockers as a first-line option for the management of uncomplicated hypertension by the ESH is a cause for concern and should be reconsidered.

## ACKNOWLEDGEMENTS

None.

### Conflicts of interest

E.S. is a current committee member of the National Institute for Health and Care Excellence (NICE) for heart failure guidelines.
